# Skin Hyperpigmentation in Coronavirus Disease 2019 Patients: Is Polymyxin B the Culprit?

**DOI:** 10.3389/fphar.2020.01304

**Published:** 2020-09-03

**Authors:** Cuicui Lu, Ning Hou

**Affiliations:** Department of Pharmacy, Shandong Provincial Hospital Affiliated to Shandong First Medical University, Jinan, China

**Keywords:** coronavirus disease 2019, polymyxin, skin hyperpigmentation, pigmentary disorder

From mid-December 2019, the outbreak of the coronavirus disease 2019 (COVID-19) in Wuhan has spread worldwide and become an emergency of major international concerns (Pascarella et al., 2020). Recently, a television report has drawn extensive attention of the public in China. Two frontline doctors of the central hospital of Wuhan, Fan Yi and Weifeng Hu, were unfortunately infected by COVID-19. Cutaneous hyperpigmentation of the two doctors was noticed in the TV interview. Many people expressed grave concerns about the pathogenesis and prognosis of their skin pigmentary disorder.

Fan Yi and Weifeng Hu were diagnosed with COVID-19 infection on January 18 and January 28, respectively. The illness progress of the two doctors was very rapid and they were transferred to the intensive care unit for further treatment. Extracorporeal membrane oxygenation (ECMO) was provided as rescue therapy for respiratory failure. Because of prolonged hospital stay, mechanical ventilation, and presence of invasive devices, some critically ill patients suffered from infections caused by multidrug-resistant (MDR) bacteria (Bassetti et al., 2018). Polymyxin B was used in these two doctors for the treatment of MDR infections. Polymyxin B is a cyclic polypeptide antibiotic that regained significant interest due to the inevitable appearance of MDR bacteria (Rigatto et al., 2019). It is even being considered as last resort therapy against some intractable gram-negative bacteria (Manchandani et al., 2018).

Acquired skin hyperpigmentation is a common dermatologic complaint, which is attributable to drugs up to 20% of all cases (Nahhas et al., 2019). Many drugs are associated with skin hyperpigmentation such as nonsteroidal anti-inflammatory agents, antihypertensive agents, antibiotics, and psychoactive agents (Giménez García and Carrasco Molina, 2018). Nephrotoxicity and neurotoxicity are common adverse reactions of polymyxin B, while skin hyperpigmentation was also reported as a side effect recently (Li et al., 2020). Reports of polymyxin B associated skin hyperpigmentation in adults were rare, unlike in neonates (Zavascki et al., 2015; Gothwal et al., 2016). Polymyxin B is mainly excreted through the kidney, dosage adjustment is required in patients with lower creatinine clearance (Zheng et al., 2018). The cumulation of polymyxin B might be a possible reason for skin hyperpigmentation in neonates due to their immature kidney function (Gothwal et al., 2016; Li et al., 2020).

Kidney involvement is prevalent in patients with COVID-19. Acute kidney injury (AKI) has been reported in approximately 20%–40% of critically ill or deceased COVID-19 patients (Ronco et al., 2020). Several mechanisms are possibly involved in COVID-19 associated AKI, including direct damage action of the virus, hemodynamic alterations, inflammatory cytokines, administration of nephrotoxic drugs, and mechanical ventilation (Gabarre et al., 2020; Martinez-Rojas et al., 2020). According to these findings, we speculate that AKI may be an important factor for polymyxin B induced pigmentary disorder in critically ill patients with COVID-19, such as Fan Yi and Weifeng Hu.

Diffuse skin hyperpigmentation was only reported in several cases (Zavascki et al., 2016). Polymyxin B induced skin hyperpigmentation was mainly on the face and neck in most cases, while other parts of the body remained unchanged throughout the course of treatment (Zavascki et al., 2015; Mattos et al., 2016; Lahiry et al., 2017). Interestingly, the density of melanocytes in the face and neck is higher than in other areas (Silpa-Archa et al., 2017). Sun exposure was excluded as cause for skin hyperpigmentation as the exposed shoulders and arms were spared.

Dermatologic manifestations are rare in COVID-19 confirmed cases. Among 1,099 hospitalized COVID-19 patients in Wuhan, only 0.2% presented with skin lesions (Guan et al., 2020). There is no report so far about COVID-19 associated skin darkening. Maculopapular eruptions, pseudochilblain, and urticarial lesions are the most common cutaneous manifestations (Kaya et al., 2020). Trunk and extremities are the main involved areas and lesions usually resolve spontaneously in a few days (Kaya et al., 2020; Recalcati, 2020). Therefore, skin hyperpigmentation is unlikely to be caused by the novel coronavirus.

In a cohort study, 60 patients treated with intravenous polymyxin B were followed up. Skin hyperpigmentation occurred in 15% of patients and the incidence was higher in darker-skinned patients than in Caucasian patients. Skin darkness was usually noted on the 3^rd^ day of treatment and decreased gradually after polymyxin B cessation (Mattos et al., 2016). According to a prospective study, the incidence of skin hyperpigmentation was 8%. Skin changes also appeared on the 3^rd^ day of treatment and the pigmentary disorder became absolutely evident after 7 days of polymyxin B treatment (Mattos et al., 2017).

Whether skin hyperpigmentation will be completely resolved after discontinuation of polymyxin B remains controversial. It was reported that only a small part of the study population survived a few months after the end of the treatment (Mattos et al., 2017). Gothwal S et al. reported one neonate restored to the original color on 45 days, but the other two neonates lost follow up (Gothwal et al., 2016). It was shown in another study that skin darkness of a 46-year-old Hispanic man improved gradually but not reversed to the baseline, even after 5 months of treatment (Knueppel and Rahimian, 2007). Fan Yi was discharged on May 9 and a partial recovery of skin color was noticed. Unfortunately, the condition of Weifeng Hu was deteriorated and he died on June 2.

The pathogenesis of polymyxin B associated skin damage could be related to histamine release and inflammatory response. Histamine is stored primarily in human mast cells and basophils, while Polymyxin B can promote the release of the stored histamine (Lassalle et al., 2003; Mattos et al., 2017). Yoshida et al. showed that histamine increased cAMP accumulation and protein kinase activity *via* H_2_ receptors, resulting in the overexpression of melanin (Yoshida et al., 2000). Lee et al. found that growth-differentiation factor-15 (GDF-15) was also involved in histamine-induced melanogenesis by increasing melanin production and chemotactic migration (Lee et al., 2012). Furthermore, epidermal Langerhans cells are antigen-presenting cells, which play an important role in chronic skin inflammation. Histologic examinations were performed in several patients who suffered from polymyxin B induced skin hyperpigmentation, and hyperplasia of Langerhans cells in epidermis was found in their skin biopsies (Mattos et al., 2017). Since histamine is also associated with inflammatory response, it is presumed that skin hyperpigmentation is a post-inflammatory effect.

So far, there has been no ideal preventive and therapeutic approaches for polymyxin B associated skin hyperpigmentation. Removal of provoking factors, laser cosmetic therapy and topical whitening agents could help recover as soon as possible. Although the outcome of therapy is not affected, skin darkness can cause mental stress and aesthetic damage. Healthcare workers should be aware of this rare adverse event, and skin color changes should be monitored frequently. Higher concentration of polymyxin B may be relevant to skin darkening in some studies (Zheng et al., 2018). Once polymyxin B induced skin hyperpigmentation was observed, it was crucial to make dose adjustment or replace it with an alternative medication regimen.

## Author Contributions

NH was responsible for the study conception and design. CL drafted the manuscript. NH revised and edited the manuscript. All authors contributed to the article and approved the submitted version.

## Conflict of Interest

The authors declare that the research was conducted in the absence of any commercial or financial relationships that could be construed as a potential conflict of interest.
